# Masquelet Technique in the Management of Segmental Bone Defect of the Ulna: A Case Report

**DOI:** 10.7759/cureus.75089

**Published:** 2024-12-04

**Authors:** Thomas E Messer, Tye Barber DO

**Affiliations:** 1 Department of Family Medicine, Broward Health Medical Center, Fort Lauderdale, USA

**Keywords:** antibiotic cement, comminuted fracture, discharge against medical advice, flexible intramedullary nail, infected hardware, masquelet technique, non-compliant patient, osteomyelitis, segmental bone defect, ulna

## Abstract

Surgeons periodically encounter challenging clinical scenarios that require them to develop nuanced management strategies to achieve the best outcome for the patient. This is especially true in medically underserved patient populations, where follow-up and proper recovery protocols are often not accomplished. In this report, we discuss the case of a 26-year-old female with a history of medical non-compliance who presented to the emergency department with signs and symptoms of surgical site infection two months following the repair of her comminuted ulna fracture caused by a gunshot wound. The decision was made to perform the Masquelet-induced membrane technique, as it is an option when confronted with segmental bone defects in patients in whom there is suspicion of noncompliance with recovery recommendations.

## Introduction

There are many strategies for addressing segmental bone defects, with the specific technique often being driven by the nature of the case at hand. Some considerations when determining the best technique include the quality of the remaining bone, with poor bone quality often necessitating additional hardware reinforcement to provide adequate strength; the size of the deformity, with small defects often able to be treated with bone grafts, bone substitutes, or through spontaneous healing; whereas extensive defects may require more complex techniques such as induced membrane, distraction osteogenesis, bone tissue engineering (BTE), and vascularized bone grafts (VBG) [1-3]. Other considerations include the patient's age, health status, and ability to undergo a multi-part procedure, the condition of the soft tissue, and the functional goals of the patient, including mobility and weight-bearing capacity.

Due to the importance of adherence to recovery recommendations following the repair of segmental defects, achieving favorable outcomes in patients in whom there is suspicion of non-compliance following surgery poses a challenge. Although somewhat counterintuitive, as the Masquelet is a two-part procedure compared to a one-part procedure such as the VBG, in cases where the patient is deemed to be high-risk for non-compliance following surgery, the Masquelet may be the best option. The first phase entails the placement of a flexible nail surrounded by antibiotic cement. If the patient does not comply with recovery and antibiotic recommendations following surgery, hardware fixation provides greater stability of the fracture compared to other techniques that do not employ hardware fixation, with the added benefit of being able to add antibiotics to the cement in patients at high risk or with active infection.

## Case presentation

**Figure 1:**
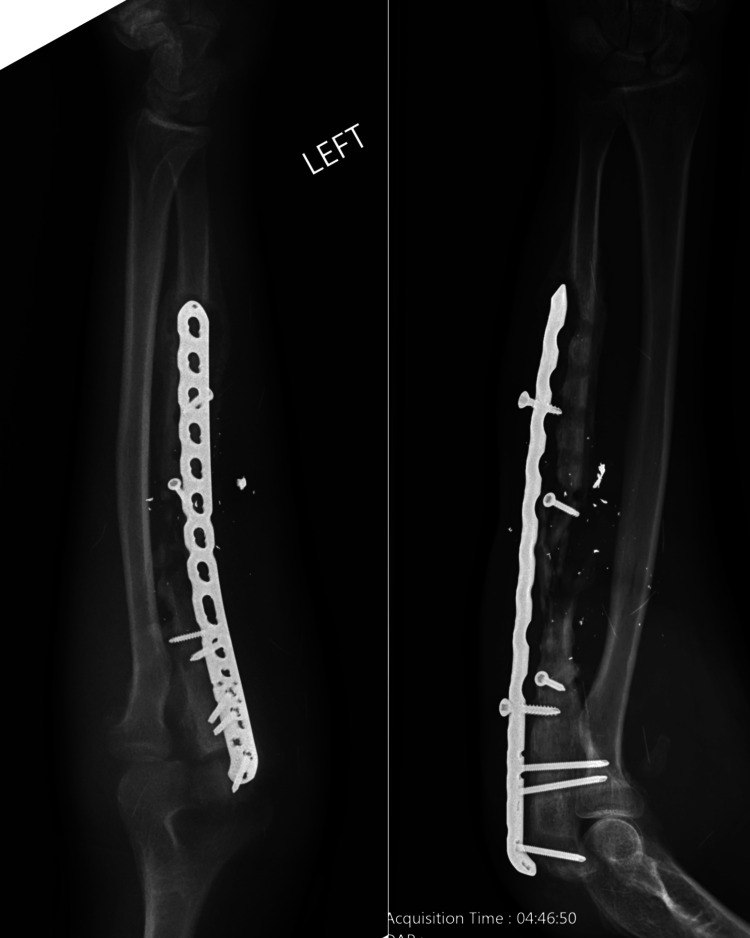
Anteroposterior and lateral views of the patient's forearm upon presentation. Note the lucency surrounding hardware, separation of the plate off the bone, and diffuse signs of inflammation.

A 26-year-old female with a history of a repaired comminuted left ulna fracture caused by a gunshot wound presents to the emergency department with signs and symptoms of surgical site infection. The patient underwent repair at an outside institution two months ago, which consisted of irrigation and debridement of the fracture site, and plate fixation to re-establish proper anatomic alignment and length. Initial imaging revealed significant osseous destruction, hardware loosening, and soft tissue edema (Figure 1). 

**Figure 2:**
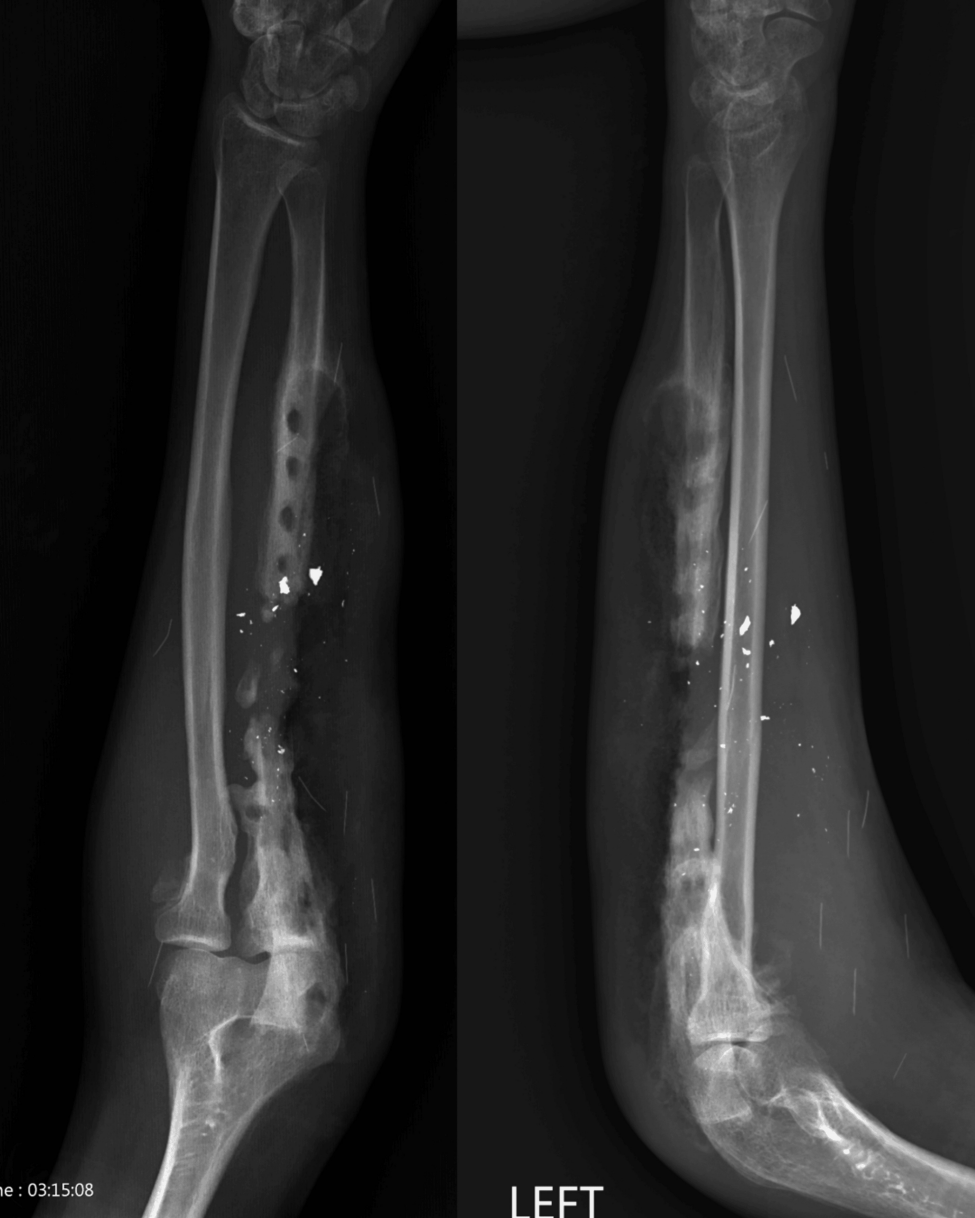
Anteroposterior and lateral views of the forearm following removal of hardware. Notice the significant defect in the ulnar shaft, osseous destruction caused by osteomyelitis, and the holes from the previous hardware. Also, note the ~10 broken needles throughout the patient’s forearm and elbow.

Following preoperative medical clearance, the patient was taken to surgery. The infected hardware was removed, and devitalized soft and osseous tissue was debrided, resulting in a 5.5-centimeter segmental defect of the ulnar shaft (Figure 2). Intraoperative cultures confirmed the presence of methicillin-resistant Staphylococcus aureus (MRSA) osteomyelitis. The wound was then covered with a wound vac, and the patient began a six-week course of IV vancomycin per the recommendation of the infectious disease service.

**Figure 3:**
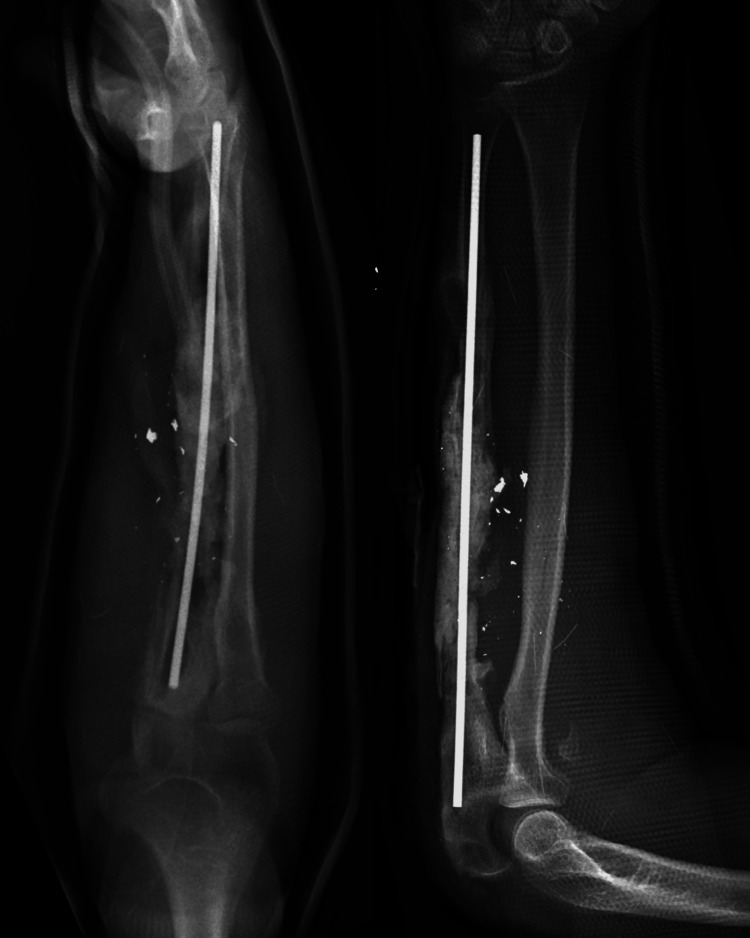
Anteroposterior and lateral views of the forearm following Masquelet's first stage. Notice the flexible nail spanning the length of the ulna, with the segmental defect surrounded circumferentially by cement.

Following the removal of hardware, the patient elected to leave the hospital with her wound vac in place. As mentioned previously, the patient had a history of illicit drug use and cited intolerable withdrawals as the motivator behind leaving the hospital despite her essentially open wound and complete lack of stability of her ulna with the hardware removed. The patient was out of the hospital for five days, before presenting to the emergency department one evening with pain and discharge from her wound. The patient then underwent another surgical debridement, followed by the first stage of the Masquelet procedure two days later (Figure 3). The procedure entailed the placement of a flexible nail fixating the proximal and distal ends of the ulna into proper anatomic alignment and length, followed by surrounding the nail circumferentially with antibiotic-impregnated cement.

Due to the extent of soft tissue damage caused by the multiple debridements, flap closure was necessary and was performed utilizing the flexor carpi ulnaris.

The patient completed her course of IV antibiotics and was discharged with instructions, along with verbal confirmation of her understanding of the need for follow-up with the orthopedic service. As of three months after discharge, she has not returned to the admitting hospital for the second stage of the procedure.

## Discussion

The Masquelet technique is a surgical method first performed in 1986 by Dr. Alain Masquelet for the treatment of large bony defects seen in non-unions and other segmental bone defects resulting from trauma, infection, tumor resection, or congenital abnormalities. The technique consists of two steps: the induction phase, which involves the placement of a cement spacer to induce a foreign-body reaction leading to the formation of a vascularized membrane around the bone defect, followed by the reconstruction phase, performed six to eight weeks later, during which the spacer is removed and a bone graft is placed into the newly formed, well-vascularized membrane covering the defect [4].

Although Masquelet himself described the use of a silicone tube as the spacer material, the predominant spacer in use today is the polymethyl methacrylate (PMMA) spacer. PMMA, a cement made from the polymerization of methyl methacrylate monomers, provides mechanical stability, serves as a temporary scaffold for bone regeneration, and can elute antibiotics if needed. PMMA first gained popularity in orthopedic surgery for its use in total joint arthroplasty due to its proven efficacy in fixating implants to bone. While PMMA is currently the most commonly used spacer, ongoing research is exploring alternative materials, including calcium sulfate, with recent studies showing a similar histologic profile, increased induced membrane thickness, and significantly greater osteoinductivity compared to PMMA [5].

The rationale of the spacer is essentially to induce a foreign body reaction resulting in the formation of a membrane. Previous trials found that the pseudo-synovial membrane formed in the first stage possessed rich vascularization, as well as the production of growth factors (VEGF, TGFβ1) and osteoinductive factors (BMP-2), which promote the maturation of the bone graft following the second phase [6]. While the Masquelet technique was the first induced membrane technique developed and remains the most well-known and widely used, variations of this technique have been made, with most variations relating to different materials or methods of creating the vascularized membrane.

The choice of the Masquelet technique in this scenario was driven by the patient’s social situation. The patient’s history of IV drug use with reused needles, leaving against medical advice (AMA) following her initial repair with poor compliance to recovery protocols and again following her hardware removal, as well as other factors that will not be disclosed to maintain patient confidentiality, raised concerns that she would leave AMA shortly following her procedure to be high. The reason the Masquelet technique is the best option for these patients, even if they do not return for the second phase, is that the first phase of the procedure includes the placement of hardware, providing some fixation, combined with the use of antibiotic-impregnated cement as a transient means of antibiotic delivery, either as prophylaxis or, in this case, to combat active infection - especially if the patient does not comply with their recommended antibiotic regimen. Although the second phase would provide much greater stability following the maturation of the bone graft, the fixation achieved through the initial phase is greater than that achieved through other segmental defect repair strategies in the period immediately following surgery, such as the VBG and BTE, both of which lack internal fixation.

If this patient were to leave shortly after a VBG and not adhere to antibiotic recommendations, this would be a problem as the VBG provides no hardware fixation, provides no transient antibiotic presence, creates a new nidus for infection at the bone harvesting site, as well as various possible vascular complications relating to the graft that may occur. Other large defect repair techniques, such as distraction osteogenesis and BTE, would also be inferior to the Masquelet technique in such cases, as the pins of the external fixator used in distraction osteogenesis are niduses of infection, especially with improper pin care, and the bone harvesting site for BTE creates new niduses for infection, provides no hardware fixation, and lacks internal antibiotics [1,2].

## Conclusions

The Masquelet technique is an option when encountering segmental bone defects in patients who are deemed to be at significant risk of non-adherence to recovery protocols. It provides both stability through mechanical fixation and the ability to add antibiotic-impregnated cement to combat or prevent infection.
